# Olfactory dysfunction increases progression to dementia in cognitively impaired older adults: a 12-year population-based study

**DOI:** 10.1007/s11357-025-01705-7

**Published:** 2025-05-28

**Authors:** Javier Oltra, Ingrid Ekström, Maria Larsson, Jane Yan, Giulia Grande, Erika J. Laukka

**Affiliations:** 1 Aging Research Center, Department of Neurobiology, Care Sciences and Society, Karolinska Institutet and Stockholm University, Stockholm, Sweden; 2 Gösta Ekman Laboratory, Department of Psychology, Stockholm University, Stockholm, Sweden; 3 Stockholm Gerontology Research Center, Stockholm, Sweden

**Keywords:** Dementia, Olfaction, Population-based study, Preclinical marker

## Abstract

Olfactory deficits are hypothesized to precede cognitive decline and be independently associated with future dementia. Conversely, the concurrency of cognitive and olfactory impairments is expected to represent an advanced stage, associated with shorter time to diagnosis. Limited research has examined the association of isolated and concurrent cognitive and olfactory impairments with incident dementia. We aimed to estimate the 12-year dementia hazard for cognitive impairment no dementia (CIND), olfactory dysfunction (OD), and their combination in a population-based cohort of older adults. We classified 2406 participants from the Swedish National Study on Aging and Care in Kungsholmen (SNAC-K) based on baseline CIND and OD. Dementia hazard was estimated with Cox regressions for the whole period and two timeframes (baseline to 6-year follow-up and 6- to 12-year follow-up), and time until receiving a diagnosis via Laplace regressions. CIND+OD was associated with increased hazard ratio (HR) of dementia over 6 years (HR 11.38; 95% CI 6.70, 19.32; *p* < 0.001), higher for amnestic CIND+OD (HR 22.23; 95% CI 11.79, 41.90; *p* < 0.001). Isolated CIND was associated with dementia closest to baseline (HR 3.38; 95% CI 1.75, 6.49; *p* < 0.001), while isolated OD was associated with dementia closest (HR 2.56; 95% CI 1.48, 4.43; *p* < 0.001) and furthest (HR 2.12; 95% CI 1.41, 3.19; *p* < 0.001) to baseline. CIND+OD received their dementia diagnosis 3 years earlier. This study demonstrated that individuals with both cognitive and olfactory impairments have a higher short-term risk of progression to dementia and that OD may be a valuable early marker of dementia on its own.

**Supplementary Information:**

The online version contains supplementary material available at 10.1007/s11357-025-01705-7.

## Introduction

Dementia, especially Alzheimer’s disease (AD), is characterized by a long prodromal phase [1]. Olfactory dysfunction (OD) has emerged as an early dementia sign [2–4]. However, several questions remain regarding the interplay between olfactory and other predementia markers.

Olfactory markers have been postulated to stratify individuals at different risk levels for dementia. In previous studies, a worse odor identification (OID) ability predicted progression from cognitive impairment (CI) to AD dementia in older adults [5]. Furthermore, better OID was associated with a reduced likelihood of transition from CI to all-type dementia and with cognitive maintenance in cognitively unimpaired individuals [6–8]. More recently, combining cognitive screening and OID tests has shown to be comparable to amyloid PET scans in estimating progression to CI or dementia [9].

These findings suggest that olfactory assessment could be a simple and cost-efficient tool for monitoring patients in primary care, identifying those at higher risk of dementia for referral to memory clinics, and selecting participants for clinical trials. However, there is a research gap in exploring the association of isolated and concurrent cognitive and olfactory impairments with future dementia. In this regard, an early study showed that older individuals with CI and OD showed an almost two-fold increased risk of progression to all-type dementia over 3.5 years compared to individuals with only CI [10]. Moreover, it has been hypothesized that olfactory deficits may appear early in the dementia course preceding memory deterioration and CI [2]. Considering the suggested sequence of pre-dementia events for cognitive and olfactory impairments, one could hypothesize that their concurrency would be related to incipient dementia in the short term, whereas presenting OD alone would be an early pre-dementia marker.

To increase our understanding of the contribution of OD in early dementia detection, in this epidemiological study, we aimed to examine the association between isolated and combined CI and OD with progression to dementia across 12 years in a large community-based cohort of older adults. We further focused on exploring these associations in two timeframes, closer (0–6 years) and further away (6–12 years) from baseline. Additionally, we aimed to evaluate the impact of memory impairment, *APOE* ε4 status, age, and sex on these relationships.

## Methods

### Study design and participants

**Fig. 1:**
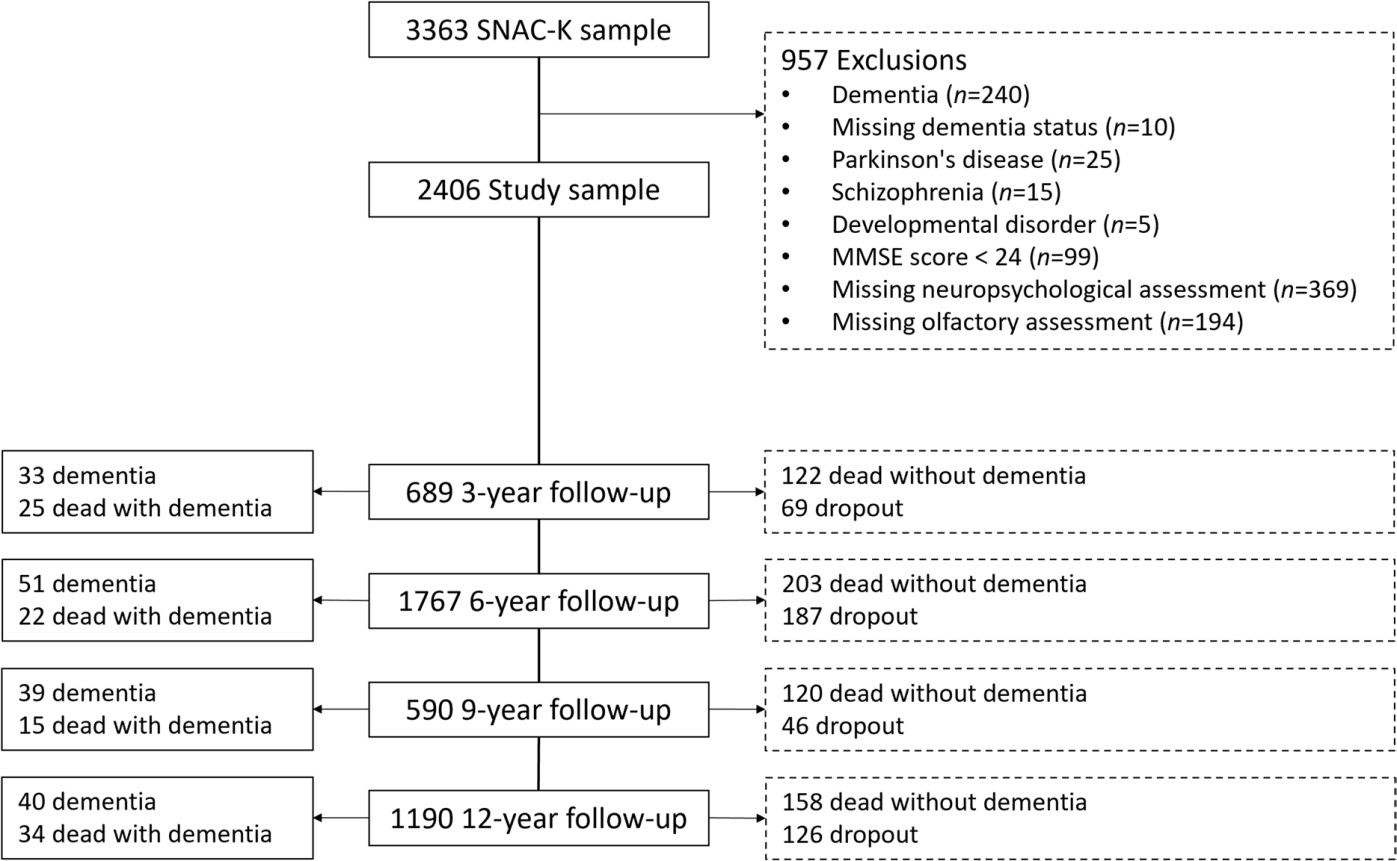
Flow chart of study participation. MMSE, Mini-Mental State Examination; SNAC-K, Swedish National Study on Aging and Care in Kungsholmen

The Swedish National Study on Aging and Care in Kungsholmen (SNAC-K) is a population-based study consisting of 3363 older adults, recruited from 2001 to 2003, who belonged to predefined age strata and were randomly selected from the Kungsholmen area in central Stockholm [11]. For the current study, we selected 2406 participants with available baseline cognitive and olfactory data (Fig. 1). All participants underwent a nurse interview, a medical examination, and a neuropsychological assessment at baseline. At follow-up, participants in the younger groups (60, 66, 72 years old) underwent re-examinations every 6 years and every 3 years after turning 78. For the older age groups, 78 years and over, participants were called back every 3 years.

All parts of SNAC-K were approved by the ethical committee at Karolinska Institutet or the Regional Ethical Review Board in Stockholm. In compliance with the Declaration of Helsinki, all participants or their appropriate surrogates provided written informed consent.

### Neuropsychological assessment

Participants underwent a comprehensive neuropsychological assessment administered by psychologists addressing five cognitive domains, as follows:

Episodic memory: The episodic memory task tailored for SNAC-K consisted of a list of 16 unrelated concrete nouns presented both orally and visually [12]. After their presentation, the participants had 2 min for free oral recall. The free recall score, computed as the number of correctly recalled words, was used for CIND classification.

Executive function: The executive function task was the trail making test-B (TMT-B), which required participants to connect 13 encircled digits and letters in an alternating numerical and alphabetical order (1-A, 2-B, 3-C, etc.) [13]. The TMT-B score was computed as the number of correct connections.

Language: Language tasks consisted of two letter fluency (F and A) and two category fluency (animals and professions) tasks [13]. Participants were given 60 s to orally generate as many words as possible. The scores consisted of the number of words generated in one minute for each one of the tasks.

Visuospatial abilities: The visuospatial abilities task consisted of a 10-item version of the Shepherd–Metzler mental rotation test [14, 15]. In each item, the participant should decide, within 45 s, which one of the three rotated figures presented matched a target figure. The score was computed as the number of correctly selected figures.

Perceptual speed: The perceptual speed tasks consisted of two paper-and-pencil tests. In digit cancellation, 11 rows of random digits ranging from 1 to 9 were presented and the participants should inspect the rows sequentially and cross out every digit 4 they detected [16]. In pattern comparison, the participants should sequentially judge and mark accordingly if 30 different pairs of line-segment patterns presented in two different sheets (15 pairs per sheet) were “same” or “different” [17]. The score was the number of digits correctly crossed out within 30 s for digit cancellation and the mean number of correct classifications within 30 s for the two trials for pattern comparison.

### Dementia diagnosis

All-cause dementia was diagnosed at baseline and follow-ups according to the *Diagnostic and Statistical Manual of Mental Disorders, 4 th edition* (*DSM-IV*), based on the information collected during the medical examination [18]. A preliminary diagnosis was made by a physician after a medical examination, which included a neurological examination and assessment of clinical history, drug use, and cognitive functioning. Performance on the neuropsychological assessment presented above was not used for diagnostic purposes. The cognitive evaluation used for dementia diagnosis included the Mini-Mental State Examination (MMSE) [19], the clock test [20], digit span [21], a frontal lobe task [22], and items regarding problem-solving, abstract thinking, self- and time–space orientation, and general knowledge [23]. A second physician made an independent diagnosis based on the documentation from the medical examination. In case of disagreement, the final diagnosis was made by a senior neurologist. Furthermore, the Swedish National Cause of Death Register, clinical charts, and medical records were examined to identify additional dementia cases among participants who died between two assessments and developed dementia after their last examination. The date of diagnosis was the examination date for participants receiving a dementia diagnosis in SNAC-K and the date of death for dementia cases identified using alternative sources of information.

### Operationalization of cognitive impairment no dementia (CIND)

For CIND classification, *z* scores for each test and participant were computed based on the baseline age group-specific mean and SD. The average of the relevant *z* scores was calculated when a specific domain involved more than one test (language and perceptual speed). Participants with a *z* score of 1.5 or more below the age group–specific mean in at least one cognitive domain in the absence of dementia were classified as having CIND. We further classified CIND into subtypes depending on the impairment in the memory domain (amnestic CIND, aCIND) or only in non-memory domains (non-amnestic CIND, naCIND).

### Olfactory assessment

Olfactory function was assessed with a normative OID test with high test–retest reliability, the Sniffin’ Sticks [24, 25]. The administrator asked the participants to freely identify 16 common household odors presented individually in felt-tip pens. If the participant was unable to produce a name or failed to provide the correct name for a specific odor, four written alternatives were provided (one target and three foils). The participants were instructed to select the option that best matched the presented odor. They received a score of 1 for each correct identification. In the few cases where information for an item was missing (e.g., due to administration error or allergy), they received a score of 0.25 (equivalent to the chance level). The OID score ranges from 0 to 16. OD was operationalized based on normative data as scoring < 11 [26–28].

### Covariates

Demographics (age, sex, and education as years of formal schooling) and lifestyle (smoking status as never/former or current smoking) information were collected in the baseline interview. We used mean-centered age and education in further analysis. Information on vascular risk factors (presence/absence of diabetes and hypertension), cardiovascular (presence/absence of ischemic heart disease, heart failure, or atrial fibrillation), and cerebrovascular (presence/absence of stroke) diseases was collected via the nurse interview, medical examination, medication lists, laboratory tests, and the Swedish National Patient Register [29].

Genotyping was performed on the Sequenom MassARRAY® platform at the Mutation Analysis Facility, Karolinska Institutet, and *APOE* was dichotomized into ε4 carriers vs. non-ε4 carriers.

### Statistical analysis

We used Cox regressions to estimate hazard ratios (HRs) for incident dementia between baseline and the 12-year follow-up. We used participants with unimpaired cognition and olfaction as the reference group (unimpaired), and the remaining groups indicated an impairment only in olfaction or cognition (isolated CIND and isolated OD) or in both domains (CIND+OD). We applied a basic model (adjusted for sex, age, and education) and a multi-adjusted model including smoking status, hypertension, diabetes, cardiovascular disease, stroke, and *APOE* ε4 status as additional covariates. The follow-up time was computed as years from baseline to dementia diagnosis, death without dementia, dropout without dementia, or end of follow-up. The difference in dementia hazard between impaired groups within the entire period was statistically tested by running pairwise models.

Furthermore, we ran equivalent Cox regressions after splitting the timing of dementia diagnoses into two periods, between baseline and 6-year follow-up and between 6- and 12-year follow-ups, to investigate progression to dementia closer and further from baseline. We applied Cox regressions with a time-varying coefficient [30]. Following the study design, we split the time based on the last participant who underwent the 6-year follow-up visit, 6.68 years after baseline (first period: 0.003–6.68 years, second period: 6.69–13.61 years). For the second period, participants who were diagnosed with dementia, died without dementia, and dropped out without dementia during the first period were omitted.

The proportional hazard assumption was tested using Schoenfeld’s residuals regressed against follow-up time. A deviation from the assumption was detected for CIND+OD, reflecting a weaker association after the 6-year follow-up.

In supplementary analyses, we performed interaction analyses on progression to dementia over 12 years of CIND and OD combinations for *APOE* ε4 status, age group (young-old, < 78 years; and old-old, ≥ 78 years), and sex. We performed stratified analyses when a significant interaction was detected. Moreover, we repeated the main Cox regressions examining the amnestic and non-amnestic CIND subtypes in combination with OD.

In sensitivity analyses, we accounted for death as a competing risk event by estimating the subdistribution hazard ratio (sHR) [31].

Lastly, we used Laplace regressions to estimate the time until receiving a dementia diagnosis as a function of the CIND and OD combinations [32]. We ran three different models based on the observed percentage of progression to dementia over 15 years within groups (unimpaired ≈ 5%, isolated CIND ≈ 9%, isolated OD ≈ 19%). In the first model, we assessed differences in the time until the first 5% of participants in each group developed dementia (5 th percentile survival time [ST]; reference group: unimpaired). In the subsequent models, we assessed differences in the median time until the first 9% of participants in each impaired group (9 th percentile ST; reference group: isolated CIND) and until 19% of participants in isolated OD and CIND+OD groups developed dementia (19 th percentile ST; reference group: isolated OD). We ran pairwise comparisons to test differences between impaired groups.

We used Stata version 18 (StataCorp LLC) for all the analyses.

## Results

The mean age at baseline of the total sample was 71.8 years (SD 9.7), 60.8% were females, and the average years of formal schooling was 12.3 (SD 4.2). Participants with CIND+OD scored lower on the MMSE and the OID task, and were more likely to be older, have fewer years of education, and have hypertension, history of heart disease, and stroke (Table 1).

**Table 1:** Baseline population characteristics by CIND and OD combinations (*n* = 2406)

	Unimpaired (*n* = 1403)	Isolated CIND (*n* = 326)	Isolated OD (*n* = 474)	CIND+OD (*n* = 203)
Baseline age, years	69.2 (8.6)	70.1 (8.7)	77.7 (10.1)	79.1 (8.5)
Sex				
Female	860 (61.3)	220 (67.5)	252 (53.2)	130 (64.0)
Male	543 (38.7)	106 (32.5)	222 (46.8)	73 (36.0)
Education, years	13.1 (4.2)	11.5 (4.3)	11.6 (4.2)	9.6 (3.3)
CIND subtypes				
Amnestic	NA	81 (24.8)	NA	58 (28.6)
Non-amnestic	NA	244 (74.8)	NA	138 (68.0)
Missing	NA	1 (0.3)	NA	7 (3.4)
MMSE	29.3 (1.0)	28.8 (1.3)	28.7 (1.3)	27.8 (1.7)
Current smoking status				
Non-smoker	1188 (84.7)	260 (79.8)	417 (88.0)	168 (82.8)
Smoker	208 (14.8)	62 (19.0)	55 (11.6)	34 (16.7)
Missing	7 (0.5)	4 (1.2)	2 (0.4)	1 (0.5)
Hypertension	647 (46.1)	169 (51.8)	284 (59.9)	122 (60.1)
Missing	3 (0.2)	NA	NA	NA
Diabetes	96 (6.8)	46 (14.1)	46 (9.7)	26 (12.8)
Cardiovascular disease	197 (14.0)	62 (19.0)	147 (31.0)	73 (36.0)
Stroke	41 (2.9)	25 (7.7)	25 (5.3)	23 (11.3)
OID score	13.3 (1.4)	13.0 (1.4)	7.9 (2.1)	7.6 (2.2)
*APOE* ε4 status				
ε4-carriers	376 (26.8)	85 (26.1)	140 (29.5)	66 (32.5)
non ε4-carriers	973 (69.4)	215 (66.0)	305 (64.3)	114 (56.2)
Missing	54 (3.8)	26 (8.0)	29 (6.1)	23 (11.3)

*CIND* cognitive impairment no dementia, *MMSE* Mini-Mental State Examination, *OD* olfactory dysfunction, *OID* odor identification

Data are mean (SD) or *n* (%) by CIND and OD combinations

During the 12-year follow-up (mean 8.2 years, SD 4.2), 603 participants died without dementia, 221 dropped out without dementia after the baseline examination, 207 dropped out without dementia after completing at least one follow-up examination, and 259 incident dementia cases were identified. Participants with isolated OD and CIND+OD were overrepresented among those who developed dementia and died but were not more likely to drop out of the study (Supplementary Table 1).

**Fig. 2:**
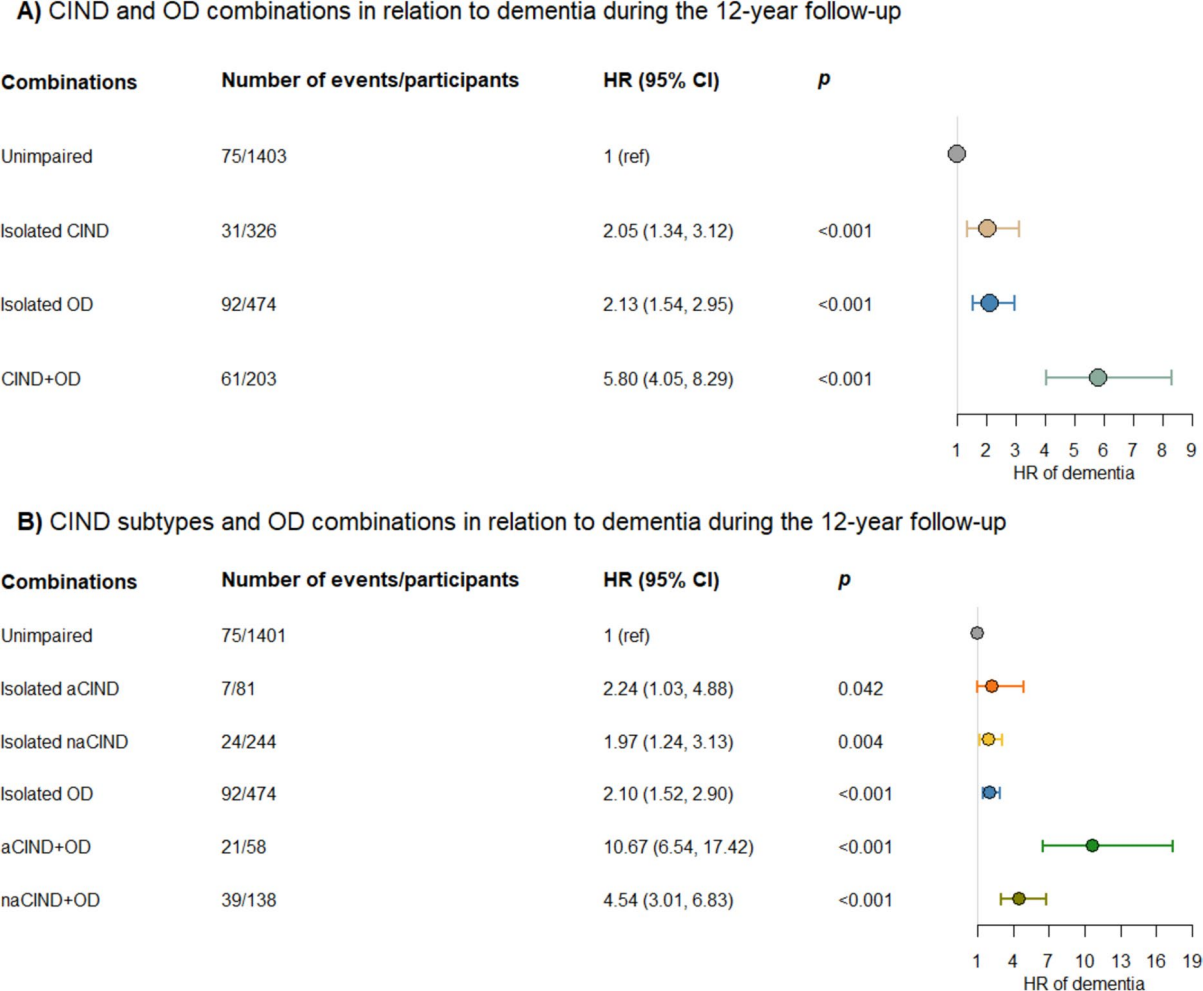
CIND and OD combinations in relation to dementia during the 12-year follow-up. **A** Results for CIND and OD combinations. **B** Results for CIND subtypes and OD combinations. HRs (95% CIs) and *p* values were obtained from the Cox regression models. Error bars indicate 95% CIs. aCIND, amnestic CIND; CIND, cognitive impairment no dementia; HR, hazard ratio; naCIND, non-amnestic CIND; OD, olfactory dysfunction

During the 12-year follow-up period, compared with unimpaired participants, the HR of dementia was 2.05 (95% CI 1.34, 3.12; *p* < 0.001) for those with isolated CIND, 2.13 (95% CI 1.54, 2.95; *p* < 0.001) for those with isolated OD, and 5.80 (95% CI 4.05, 8.29; *p* < 0.001) for those with CIND+OD (Fig. 2A). The HR was two to three times and significantly higher for participants with CIND+OD compared with those with isolated CIND (*p* < 0.001) and isolated OD (*p* < 0.001). The HR was not significantly different between participants with isolated CIND and isolated OD (*p* = 0.626). In the multi-adjusted models, the HRs were generally slightly attenuated (Supplementary Table 2).

In the first time period, between baseline and 6-year follow-up, 129 participants were diagnosed with dementia. During this period, compared with unimpaired participants, the HR was 3.38 (95% CI 1.75, 6.49; *p* < 0.001) for those with isolated CIND, 2.56 (95% CI 1.48, 4.43; *p* < 0.001) for those with isolated OD, and 11.38 (95% CI 6.70, 19.32; *p* < 0.001) for those with CIND+OD (Table 2). The HR was three to five times and significantly higher for participants with CIND+OD compared with those with isolated CIND (*p* < 0.001) and isolated OD (*p* < 0.001), and it was not significantly different between isolated CIND and isolated OD (*p* = 0.406). In the multi-adjusted models, the HRs were generally slightly attenuated (Supplementary Table 3).

**Table 2:** CIND and OD combinations in relation to dementia during two timeframes (baseline to 6-year follow-up and 6- to 12-year follow-up)

	Dementia (0–6 years)			Dementia (6–12 years)		
	Number of events/participants	HR (95% CI)	*p*	Number of events/participants	HR (95% CI)	*p*
Unimpaired	21/1403	1 (ref)		54/1007	1 (ref)	
Isolated CIND	16/326	**3.38 (1.75, 6.49)**	** < 0.001**	15/195	1.47 (0.82, 2.61)	0.195
Isolated OD	41/474	**2.56 (1.48, 4.43)**	** < 0.001**	51/270	**2.12 (1.41, 3.19)**	** < 0.001**
CIND+OD	51/203	**11.38 (6.70, 19.32)**	** < 0.001**	10/67	1.88 (0.94, 3.77)	0.074
CIND subtypes						
Unimpaired	21/1401	1 (ref)		54/1006	1 (ref)	
Isolated aCIND	2/81	2.19 (0.51, 9.37)	0.290	5/44	2.24 (0.89, 5.63)	0.087
Isolated naCIND	14/244	**3.55 (1.80, 7.03)**	** < 0.001**	10/150	1.24 (0.63, 2.46)	0.529
Isolated OD	41/474	**2.48 (1.43, 4.30)**	**0.001**	51/270	**2.11 (1.40, 3.17)**	** < 0.001**
aCIND+OD	18/58	**22.23 (11.79, 41.90)**	** < 0.001**	3/17	2.93 (0.91, 9.45)	0.071
naCIND+OD	32/138	**8.57 (4.82, 15.25)**	** < 0.001**	7/49	1.63 (0.73, 3.67)	0.235

*aCIND* amnestic CIND, *CIND* cognitive impairment no dementia, *HR* hazard ratio, *naCIND* non-amnestic CIND, *OD* olfactory dysfunction

HRs (95% CIs) and *p* values were obtained from the Cox regression models with a time-varying coefficient. Model adjusted for age, sex, and years of education

Significant results in bold

In the second time period, between 6- and 12-year follow-ups, 130 participants were diagnosed with dementia. During this period, compared with unimpaired participants, the HR of dementia was 1.47 (95% CI 0.82, 2.61; *p* = 0.195) for those with isolated CIND, 2.12 (95% CI 1.41, 3.19; *p* < 0.001) for those with isolated OD, and 1.88 (95% CI 0.94, 3.77; *p* = 0.074) for those with CIND+OD (Table 2). The HR was not significantly different between participants with isolated CIND compared with those with isolated OD (*p* = 0.203) and CIND+OD (*p* = 0.770), nor between those with isolated OD compared with those with CIND+OD (*p* = 0.851). In the multi-adjusted models, the HRs were generally slightly attenuated (Supplementary Table 3). The main difference was that the HR was significant for those with CIND+OD, while the association was still weaker than for the first period.

The results from the Cox regressions for CIND subtypes in combination with OD revealed that, during the 12-year follow-up period, participants with aCIND+OD (HR 10.67; 95% CI 6.54, 17.42; *p* < 0.001) had a 4.8-fold higher HR than those with isolated aCIND (HR 2.24; 95% CI 1.03, 4.88; *p* = 0.042), and that participants with naCIND+OD (HR 4.54; 95% CI 3.01, 6.83; *p* < 0.001) had a 2.6-fold higher HR than those with isolated naCIND (HR 1.97; 95% CI 1.24, 3.13; *p* = 0.004; Fig. 2B). During the period between baseline and 6-year follow-up, participants with aCIND+OD (HR 22.23; 95% CI 11.79, 41.90; *p* < 0.001) had a 10.2-fold higher HR than those with isolated aCIND (HR 2.19; 95% CI 0.51, 9.37; *p* = 0.290) and participants with naCIND+OD (HR 8.57; 95% CI 4.82, 15.25; *p* < 0.001) had a 2.4-fold higher HR than those with isolated naCIND (HR 3.55; 95% CI 1.80, 7.03; *p* < 0.001; Table 2). Between 6- and 12-year follow-ups, the HRs were largely attenuated, and CIND subtypes either isolated or with OD were not significantly associated with incident dementia (Table 2). In the multi-adjusted models, the HRs were generally slightly attenuated (Supplementary Tables 2 & 3). The main difference was that, for the second period, the HR was significant for participants with isolated aCIND, while the association was still weaker than for the first period.

Supplementary analyses revealed a significant interaction between age groups, isolated OD, and dementia diagnosis over 12 years in the basic model (*p* = 0.017). Young-old participants with isolated OD (HR 3.50; 95% CI 1.96, 6.27; *p* < 0.001) had a relatively higher HR than old-old participants with isolated OD (HR 1.95; 95% CI 1.34, 2.85; *p* < 0.001). The interaction remained significant in the multi-adjusted model (*p* = 0.011): young-old participants (HR 4.19; 95% CI 2.32, 7.60; *p* < 0.001), old-old participants (HR 1.92; 95% CI 1.31, 2.82; *p* < 0.001).

Sensitivity analyses treating death as a competing risk event showed similar results to those reported (Supplementary Table 4). The main difference was that the HR was not significant for participants with isolated aCIND over 12 years in the basic model.

**Fig. 3:**
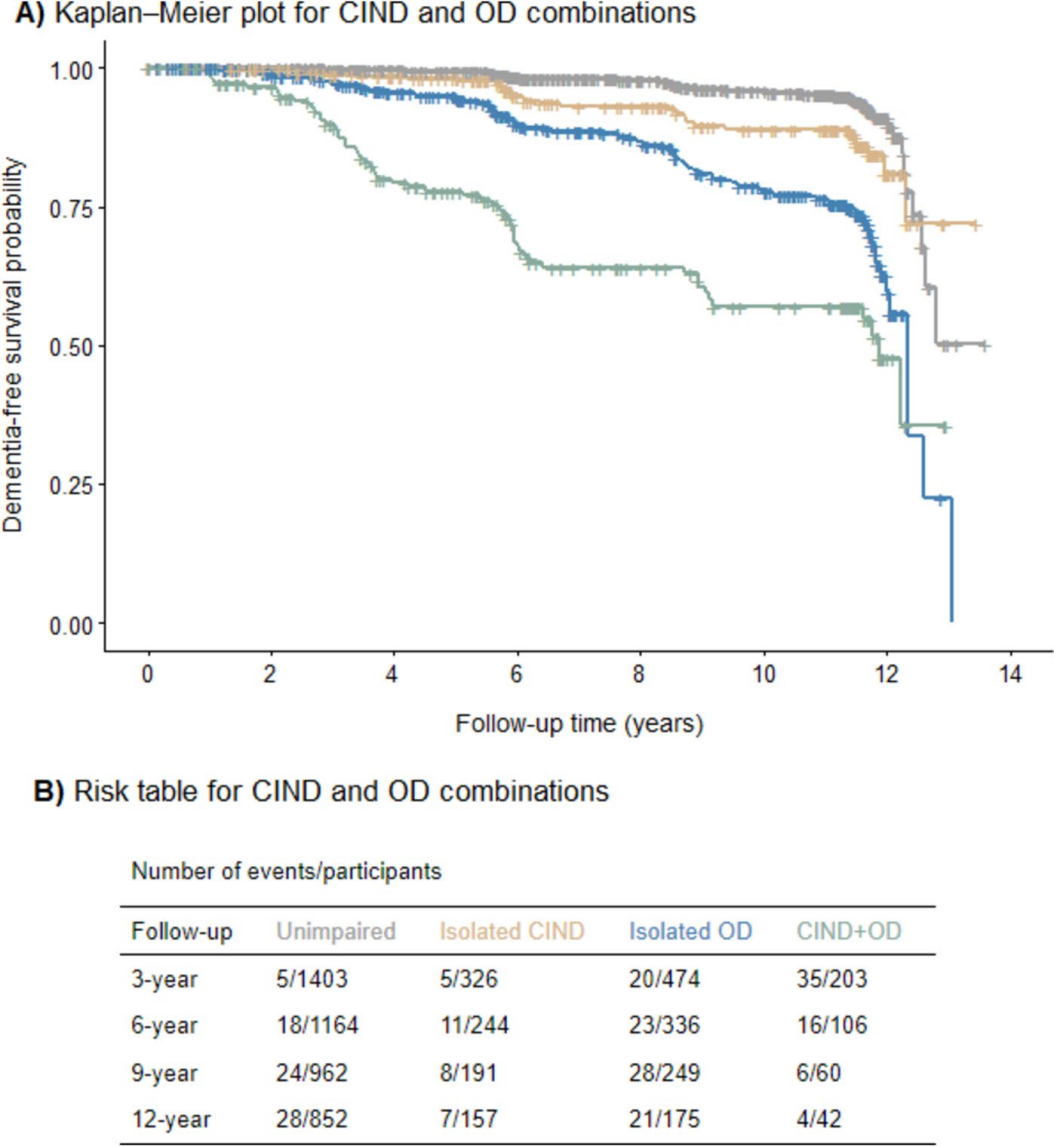
Progression to dementia for CIND and OD combinations across the whole study period. **A** Kaplan–Meier plot for CIND and OD combinations. **B** Risk table for CIND and OD combinations. CIND**,** cognitive impairment no dementia; OD, olfactory dysfunction

Participants with CIND+OD reached a lower survival rate within the first 6 years after baseline (Fig. 3). Results from the Laplace regressions are displayed in Table 3. The first model estimated that 5% of participants with CIND+OD converted to dementia almost 5 years after baseline, while individuals with isolated CIND and isolated OD reached the same proportion of incident dementia after around 8 years. There was no statistically significant difference between isolated OD and isolated CIND in time in reaching 5% of incident dementia cases. The second model resulted in slightly longer STs for 9% of progression to dementia; participants with CIND+OD reached such a percentage of progression after 5 years and isolated CIND and isolated OD after around 9 years. The third model estimated that 19% of participants with CIND+OD converted to dementia around 8 years after baseline, while those with isolated OD reached the same proportion of dementia cases after almost 11 years. In the multi-adjusted model, the STs were longer; moreover, participants with isolated CIND did not differ from unimpaired individuals in the time to reach 5% of incident dementia.

**Table 3:** Estimated survival times for CIND and OD combinations

	ST (95% CI)	*p*	Pairwise comparisons	*p*
Basic model				
5 th percentile ST				
Unimpaired (ref)	9.65 (8.81, 10.48)		Isolated CIND vs. isolated OD	0.063
Isolated CIND	**7.63 (5.41, 9.85)**	**0.004**	**Isolated CIND vs. CIND+OD**	** < 0.001**
Isolated OD	**8.30 (6.39, 10.22)**	**0.014**	**Isolated OD vs. CIND+OD**	** < 0.001**
CIND+OD	**4.84 (2.68, 6.99)**	** < 0.001**		
9 th percentile ST				
Isolated CIND (ref)	8.60 (7.48, 9.69)		**Isolated OD vs. CIND+OD**	** < 0.001**
Isolated OD	8.89 (6.46, 11.32)	0.665		
CIND+OD	**5.34 (3.02, 7.80)**	** < 0.001**		
19 th percentile ST				
Isolated OD (ref)	10.79 (9.27, 12.31)			
CIND+OD	**7.66 (4.62, 10.70)**	** < 0.001**		
Multi-adjusted model				
5 th percentile ST				
Unimpaired (ref)	10.95 (9.60, 12.31)		Isolated CIND vs. isolated OD	0.852
Isolated CIND	9.49 (6.32, 12.65)	0.111	**Isolated CIND vs. CIND+OD**	**0.043**
Isolated OD	**9.49 (6.80, 12.19)**	**0.032**	**Isolated OD vs. CIND+OD**	**0.039**
CIND+OD	**6.91 (3.42, 10.40)**	** < 0.001**		
9 th percentile ST				
Isolated CIND (ref)	10.97 (9.22, 12.72)		**Isolated OD vs. CIND+OD**	** < 0.001**
Isolated OD	10.47 (7.20, 13.75)	0.526		
CIND+OD	**7.98 (7.20, 11.17)**	** < 0.001**		
19 th percentile ST				
Isolated OD (ref)	12.33 (10.77, 13.90)			
CIND+OD	**10.02 (6.95–13.08)**	**0.002**		

*CIND* cognitive impairment no dementia, *OD* olfactory dysfunction, *ST* survival time

STs (95% CI) and *p* values were obtained from the Laplace regression models. Basic model adjusted for age, sex, and education. Multi-adjusted model additionally adjusted for current smoking status, hypertension, diabetes, cardiovascular disease, stroke, and *APOE* ε4 status

Significant results in bold

## Discussion

This population-based study of older adults showed that individuals with combined cognitive and olfactory impairments had a two to three times higher risk of developing dementia over the next 6 years compared to those only presenting impairment in one modality. The results suggest that this combination is associated with a higher progression to dementia within the next few years, and the association was even more pronounced in individuals with amnestic CI. Individuals with either isolated cognitive or olfactory impairments displayed a comparable elevated dementia risk over the first 6 years after examination, whereas individuals with isolated smell impairment displayed a reliably higher dementia hazard across the whole 12-year follow-up interval.

Isolated CI was associated with a higher hazard of dementia, which was most pronounced within the first 6 years and in individuals with the amnestic form. Participants with isolated CI displayed 9.5% dementia progression over the whole period, while those with combined cognitive and olfactory impairments displayed 30% dementia progression. These observations align with previous studies suggesting that the presence of objective CI may be insufficient to capture the complexity of predementia stages [33]. Other markers, such as olfactory deficits, could enhance the identification of specific groups with a higher likelihood of dementia progression.

Having isolated OD was associated with a more than two times increased hazard of dementia over 12 years. Importantly, this association remained stable in the period closest and furthest to baseline. This finding is in line with previous population-based studies, showing that the worse olfactory performance is associated with future dementia [3–5, 34, 35]. Notably, we observed this relationship in a group of individuals classified as cognitively unimpaired using a comprehensive neuropsychological assessment, whereas prior reports applied shorter cognitive screening tests to exclude CI at baseline [3, 4, 34]. The detected relation between isolated OD and incident dementia over 12 years reinforces its status as an early predementia marker, highlighting its independence of displaying concurrent CI. This finding is especially relevant since a crucial issue is that once objective CI is evident, the disease progression is already quite far gone for those where the cognitive symptoms are due to AD [1].

Those individuals classified as CI with OD displayed a three times higher hazard of dementia across 12 years compared with those having isolated CI. After splitting the time to incident dementia, the relationship was more pronounced within the first 6 years from baseline examination. Our findings align with prior reports of increased dementia progression for the concurrence of cognitive and olfactory impairments over shorter follow-ups in clinical and population-based settings [10, 36]. Although, in the multi-adjusted model, their concurrency was significantly associated with a higher hazard of dementia between 6- and 12-year follow-ups, it remained at the same level of isolated OD and was no longer significant after considering death as a competing risk event. Thus, we expand previous knowledge by circumscribing their incremented joint predictive value to a short term.

Next, we explored the potential modifying effects of *APOE* ε4 status, age, and sex. We found that for those individuals with isolated OD, the young-old group showed a higher hazard of dementia over 12 years than the old-old. This finding strengthens the notion that special attention should be given to olfactory deficits at younger old age [3].

The hazard associated with CI subtypes was higher for those having the memory domain affected, especially in combination with OD. When having OD, amnestic individuals had more than a doubled risk of developing dementia than those with non-amnestic impairment. The association was more pronounced within the first 6 years after baseline, reaching a 22.2-fold increased risk for amnestic CI with OD, nearly three times higher than for those with memory preserved. Our findings are consistent with previous studies reporting a higher progression to dementia in amnestic CI with olfactory deficits in population-based and clinical settings [10, 37].

Cognitive and olfactory impairments are also associated with decreased survival [38–40]. However, sensitivity analyses treating death as a competing event produced similar results.

A further point of interest was whether the importance of cognitive and olfactory impairments varies at different predementia stages. Olfactory deficits have been suggested to precede cognitive decline and impairment [2], which is supported by evidence from population-based and community-based studies [10, 41–44]. These functional changes are expected to accompany pathological events in the brain, characterized by amyloid deposition and tau formation in olfactory-related brain areas, potentially leading to neurodegeneration [2]. In this context, one could argue that isolated OD would be a long-standing predictor of dementia, while once CI concurs, it predicts dementia in the short term. In our cohort, it could be expected that those classified as isolated OD later developed CI as an intermediate predementia stage [8]. We found that having CI with OD accelerated the onset of dementia by 3 years compared to their isolated forms.

Overall, our findings are in line with the hypothesis of olfactory-first impairment in predementia stages. In addition, they highlight its relevance as an indicator of more imminent dementia progression when accompanied by CI. Further studies should address whether combining cognitive and olfactory trajectories improves dementia detection, as previous single-modality studies suggested [18, 43]. They should also investigate alternative pathways not accompanied by olfactory deficits, as suggested, for example, by our results showing that isolated CI is still related to future dementia. This approach could help protect against false positives. In this sense, better OID performance has been previously associated with a higher likelihood of transition from CI back to normal [8].

The main strength of this study is that it includes a combination of a broad neuropsychological assessment and OID testing in a large sample with a long follow-up interval involving thorough dementia assessment. The main limitation is that due to the clinical nature of dementia diagnosis, we could not perform split analyses by dementia subtypes. Moreover, the generalizability could be limited because of the participants’ profile (relativity healthy and fit, predominantly high socioeconomic standing, and mainly Swedish-born).

It is important to note that the sample size might have limited the ability to capture events (dementia diagnoses) when subtyping and splitting the timing of dementia diagnoses, especially for aCIND. Although the observed data and statistical findings reinforce the potential role of OID tasks in identifying amnestic cognitively impaired individuals at a higher short-term risk of dementia, interpretations should be cautious. Future multi-sample studies could help overcome this limitation.

OID performance relies in part on declarative memory processes [45, 46], raising the possibility that the observed olfactory impairment in individuals with aCIND reflects, at least partially, underlying deficits in verbal and semantic memory. This overlap is important to consider when interpreting the results in this group. However, our findings support the role of OD as a distinct early marker of dementia and clearly demonstrate the added value of assessing olfactory impairment in addition to memory impairment for detecting individuals with a high risk of progressing to dementia in the short term. Further studies are warranted to quantify shared and unique contributions of olfactory and memory deficits in dementia risk.

This study demonstrates the added advantage of combining cognitive and olfactory testing to identify people with increased dementia risk. The results suggest that CI accompanied by OD is a signal of incipient dementia in the coming years, especially for those with the memory domain affected. Furthermore, our findings reaffirm the robustness of OD as a long-standing early marker of dementia on its own. Our results support the notion that including an OID test in predementia screening could be a cost-efficient approach for early dementia detection in clinical settings and selection of participants for clinical trials.

## Supplementary Information

Below is the link to the electronic supplementary material.

Supplementary file1 (DOCX 35.2 KB)

## Data Availability

Data can be made available on reasonable request and approval by the SNAC-K data management and maintenance committee (snac-k.se).
