# Supplementary material for: Olfactory dysfunction increases progression to dementia in cognitively impaired older adults: a 12-year population-based study
Source: GeroScience. 2025 May 28;48(1):591–603. doi: 10.1007/s11357-025-01705-7 (PMC12972283; doi:10.1007/s11357-025-01705-7)
Supplement: Supplementary file 1 — Supplementary file1 (DOCX 35.2 KB) [file 11357_2025_1705_MOESM1_ESM.docx]

**Supplementary Table 1** Percentage of and mean time to dementia, death, and drop out by CIND and OD combinations

|  | **Percentage of incident dementia** | **Mean time to dementia** | **Percentage of death** | **Mean time to death** | **Percentage of drop out** | **Mean time to drop out** |
| --- | --- | --- | --- | --- | --- | --- |
| Unimpaired | 5.3% | 8.9 years | 19.2% | 6.4 years | 18.3% | 3.0 years |
| Isolated CIND | 9.5% | 7.5 years | 23.0% | 6.0 years | 23.3% | 2.8 years |
| Isolated OD | 19.4% | 7.5 years | 37.3% | 5.9 years | 13.9% | 2.6 years |
| CIND+OD | 30.0% | 4.8 years | 39.9% | 5.2 years | 14.3% | 1.6 years |

*CIND* cognitive impairment no dementia, *OD* olfactory dysfunction

**Supplementary Table 2** CIND and OD combinations in relation to dementia during the 12-year follow-up after adjustment for multiple confounders

|  | | **Dementia (0**–**12 years)** | | |
| --- | --- | --- | --- | --- |
|  | | **Number of events/ participants** | **HR (95% CI)** | ***p*** |
|  | Unimpaired | 73/1340 | 1 (ref) |  |
|  | Isolated CIND | 29/296 | **1.89 (1.22, 2.93)** | **0.005** |
|  | Isolated OD | 89/443 | **2.16 (1.56, 3.00)** | **<0.001** |
|  | CIND+OD | 55/180 | **4.97 (3.40, 7.27)** | **<0.001** |
| CIND subtypes | |  |  |  |
|  | Unimpaired | 73/1338 | 1 (ref) |  |
|  | Isolated aCIND | 7/71 | **2.66 (1.21, 5.84)** | **0.015** |
|  | Isolated naCIND | 22/225 | **1.70 (1.04, 2.76)** | **0.033** |
|  | Isolated OD | 89/443 | **2.13 (1.53, 2.95)** | **<0.001** |
|  | aCIND+OD | 20/52 | **8.99 (5.40, 14.98)** | **<0.001** |
|  | naCIND+OD | 34/122 | **3.76 (2.42, 5.84)** | **<0.001** |

*aCIND* amnestic CIND, *CIND* cognitive impairment no dementia, *HR* hazard ratio, *naCIND* non-amnestic CIND, *OD* olfactory dysfunction

HRs (95% CIs) and *p* values were obtained from the Cox regression models. Model adjusted for age, sex, years of education, smoking status, hypertension, diabetes, cardiovascular diseases, stroke, and *APOE* ε4 status

Significant results in bold

**Supplementary Table 3** CIND and OD combinations in relation to dementia during two timeframes (baseline to 6-year follow-up and 6- to 12-year follow-up) after adjustment for multiple confounders

|  | | **Dementia (0–6 years )** | | | **Dementia (6–12 years )** | | |
| --- | --- | --- | --- | --- | --- | --- | --- |
|  | | **Number of events/ participants** | **HR (95% CI)** | ***p*** | **Number of events/ participants** | **HR (95% CI)** | ***p*** |
|  | Unimpaired | 20/1340 | 1 (ref) |  | 53/989 | 1 (ref) |  |
|  | Isolated CIND | 14/296 | **3.00 (1.50, 6.00)** | **0.002** | 15/189 | 1.50 (0.84, 2.70) | 0.174 |
|  | Isolated OD | 38/443 | **2.58 (1.47, 4.54)** | **0.001** | 51/263 | **2.15 (1.43, 3.24)** | **<0.001** |
|  | CIND+OD | 45/180 | **9.27 (5.25, 16.35)** | **<0.001** | 10/66 | **2.15 (1.07, 4.30)** | **0.031** |
| CIND subtypes | |  |  |  |  |  |  |
|  | Unimpaired | 20/1338 | 1 (ref) |  | 53/988 | 1 (ref) |  |
|  | Isolated aCIND | 2/71 | 2.69 (0.62, 11.63) | 0.185 | 5/43 | **2.57 (1.01, 6.58)** | **0.049** |
|  | Isolated naCIND | 12/225 | **2.95 (1.42, 6.12)** | **0.004** | 10/146 | 1.24 (0.62, 2.46) | 0.546 |
|  | Isolated OD | 38/443 | **2.50 (1.42, 4.41)** | **0.001** | 51/263 | **2.14 (1.42, 3.22)** | **<0.001** |
|  | aCIND+OD | 17/52 | **19.39 (9.94, 37.81)** | **<0.001** | 3/17 | 2.69 (0.83, 8.73) | 0.100 |
|  | naCIND+OD | 27/122 | **6.37 (3.39, 11.97)** | **<0.001** | 7/48 | 1.96 (0.87, 4.41) | 0.103 |

*aCIND* amnestic CIND, *CIND* cognitive impairment no dementia, *HR* hazard ratio, *naCIND* non-amnestic CIND, *OD* olfactory dysfunction

HRs (95% CIs) and *p* values were obtained from the Cox regression models with a time-varying coefficient. Model adjusted for age, sex, years of education, smoking status, hypertension, diabetes, cardiovascular diseases, stroke, and *APOE* ε4 status

Significant results in bold

**Supplementary Table 4** CIND and OD combinations in relation to dementia during the 12-year follow-up considering death without a dementia diagnosis as a competing risk event

|  | | **Basic model** | | | **Multi-adjusted model** | | |
| --- | --- | --- | --- | --- | --- | --- | --- |
|  | | **Number of events/ participants** | **sHR (95% CI)** | ***p*** | **Number of events/ participants** | **sHR (95% CI)** | ***p*** |
|  | Unimpaired | 75/1403 | 1 (ref) |  | 73/1340 | 1 (ref) |  |
|  | Isolated CIND | 31/326 | **1.76 (1.16, 2.68)** | **0.008** | 29/296 | 1.74 (1.13, 2.69) | 0.012 |
|  | Isolated OD | 92/474 | **2.19 (1.56, 3.06)** | **<0.001** | 89/443 | **2.18 (1.55, 3.07)** | **<0.001** |
|  | CIND+OD | 61/203 | **4.00 (2.71, 5.90)** | **<0.001** | 55/180 | **3.45 (2.27, 5.26)** | **<0.001** |
| CIND subtypes | |  |  |  |  |  |  |
|  | Unimpaired | 75/1401 | 1 (ref) |  | 73/1338 | 1 (ref) |  |
|  | Isolated aCIND | 7/81 | 1.86 (0.87, 3.97) | 0.108 | 7/71 | **2.14 (1.01, 4.53)** | **0.048** |
|  | Isolated naCIND | 24/244 | **1.72 (1.08, 2.74)** | **0.023** | 22/225 | **1.63 (1.01, 2.65)** | **0.047** |
|  | Isolated OD | 92/474 | **2.10 (1.50, 2.95)** | **<0.001** | 89/443 | **2.11 (1.50, 2.97)** | **<0.001** |
|  | aCIND+OD | 21/58 | **8.50 (5.00, 14.46)** | **<0.001** | 20/52 | **7.73 (4.28, 13.96)** | **<0.001** |
|  | naCIND+OD | 39/138 | **3.15 (2.02, 4.91)** | **<0.001** | 34/122 | **2.58 (1.59, 4.18)** | **<0.001** |

*aCIND* amnestic CIND, *CIND* cognitive impairment no dementia, *naCIND* non-amnestic CIND, *OD* olfactory dysfunction, *sHR* subdistribution hazard ratio

sHRs (95% CIs) and *p* values were obtained from the Cox regression models. Basic model adjusted for age, sex, and years of education. Multi-adjusted model adjusted for age, sex, years of education, smoking status, hypertension, diabetes, cardiovascular diseases, stroke, and *APOE* ε4 status

Significant results in bold.
